# Persistent Salmonellosis Causes Pancreatitis in a Murine Model of Infection

**DOI:** 10.1371/journal.pone.0092807

**Published:** 2014-04-09

**Authors:** Kathleen E. DelGiorno, Jason W. Tam, Jason C. Hall, Gangadaar Thotakura, Howard C. Crawford, Adrianus W. M. van der Velden

**Affiliations:** 1 Department of Molecular Genetics and Microbiology, Stony Brook University, Stony Brook, New York, United States of America; 2 Department of Pharmacological Sciences, Stony Brook University, Stony Brook, New York, United States of America; 3 Department of Pathology, Stony Brook University, Stony Brook, New York, United States of America; 4 Center for Infectious Diseases, Stony Brook University, Stony Brook, New York, United States of America; 5 Department of Cancer Biology, Mayo Clinic, Jacksonville, Florida, United States of America; Duke University Medical Center, United States of America

## Abstract

Pancreatitis, a known risk factor for the development of pancreatic ductal adenocarcinoma, is a serious, widespread medical condition usually caused by alcohol abuse or gallstone-mediated ductal obstruction. However, many cases of pancreatitis are of an unknown etiology. Pancreatitis has been linked to bacterial infection, but causality has yet to be established. Here, we found that persistent infection of mice with the bacterial pathogen *Salmonella enterica* serovar Typhimurium (*S*. Typhimurium) was sufficient to induce pancreatitis reminiscent of the human disease. Specifically, we found that pancreatitis induced by persistent *S*. Typhimurium infection was characterized by a loss of pancreatic acinar cells, acinar-to-ductal metaplasia, fibrosis and accumulation of inflammatory cells, including CD11b^+^ F4/80^+^, CD11b^+^ Ly6C^int^ Ly6G^+^ and CD11b^+^ Ly6C^hi^ Ly6G^−^ cells. Furthermore, we found that *S*. Typhimurium colonized and persisted in the pancreas, associated with pancreatic acinar cells *in vivo*, and could invade cultured pancreatic acinar cells *in vitro*. Thus, persistent infection of mice with *S*. Typhimurium may serve as a useful model for the study of pancreatitis as it relates to bacterial infection. Increased knowledge of how pathogenic bacteria can cause pancreatitis will provide a more integrated picture of the etiology of the disease and could lead to the development of new therapeutic approaches for treatment and prevention of pancreatitis and pancreatic ductal adenocarcinoma.

## Introduction

Pancreatitis affects over 80,000 Americans every year, and, in its chronic form, is a known risk factor for the development of pancreatic ductal adenocarcinoma (PDA) [1], [2]. Acute pancreatitis ranges in severity from mild interstitial pancreatitis to a much more severe condition associated with necrosis and concomitant multi-organ failure [3]. Most patients with acute pancreatitis suffer from mild interstitial pancreatitis, but up to 20% of patients suffer from severe pancreatitis, which is often fatal [4]. Under conditions of persistent or repeated insult, acute pancreatitis can progress to chronic pancreatitis, which is an often asymptomatic condition diagnosed only after the development of complications. The incidence of biliary pancreatitis increased by 32% between 1994 and 2001, likely due to the climbing obesity rate and obesity-associated increase in the development of gallstones [5], [6]. Other established risk factors for the development of pancreatitis include excessive alcohol consumption, cigarette smoking, and genetic predisposition [6]. Even though 70% of chronic pancreatitis cases are attributed to alcohol abuse, 95% of alcoholics never develop pancreatitis. The remaining chronic pancreatitis cases are considered idiopathic in nature [1].

The etiology of pancreatitis remains incomplete. A number of studies have linked pancreatitis to bacterial infection, but causality has yet to be established [7]–[16]. Several case reports have implicated *Salmonellae* as a causative agent of pancreatitis [13], [17]–[27] and two retrospective studies of *Salmonella*-infected individuals have shown that the frequencies of hyperamylasemia and clinical pancreatitis were 50% and ranged from 28 to 62%, respectively [14], [28]. Although a prospective study of 30 patients infected with *Salmonella* found no direct evidence for the development of pancreatitis, increased levels of lipase in serum indicated a role for the pancreas in human salmonellosis [29].

*Salmonellae* are a leading cause of morbidity and mortality in humans worldwide [30]. Infections with *Salmonellae* range in severity from self-limiting gastroenteritis to typhoid fever and can lead to chronic carriage. Non-typhoidal *Salmonellae* such as *Salmonella enterica* serovar Typhimurium (*S*. Typhimurium) are a leading cause of inflammatory enterocolitis and death due to foodborne illness, and are a significant cause of invasive bacteremia in immunocompromised hosts. Typhoidal *Salmonellae* such as *Salmonella enterica* serovar Typhi (*S*. Typhi) cause systemic infections characterized by bacterial penetration of the intestinal barrier and extraintestinal dissemination to the liver and spleen, where the microorganisms survive and replicate in professional phagocytes [31], [32]. Septic shock and death can occur if infections are left untreated [33]. Much of what is known about the pathogenesis of and host response to *Salmonellae* comes from experimental infection of mice with *S*. Typhimurium, which has served as a useful model for the human disease caused by *S*. Typhi [32].

Here, we report that pancreata of mice persistently infected with *S*. Typhimurium consistently displayed inflammatory, fibrotic and epithelial responses similar to chronic pancreatitis in humans. *S*. Typhimurium colonized the pancreas throughout the course of infection, associated with pancreatic acinar cells *in vivo*, and could directly invade pancreatic acinar cells *in vitro*. Thus, *Salmonella* infection can cause pancreatitis, a known risk factor for the development of PDA.

## Materials and Methods

### Ethics Statement

All procedures and experiments using mice were approved by Institutional Animal Care and Use Committees at Stony Brook University or Mayo Clinic, and were conducted in accordance with the recommendations outlined in the Guide for the Care and Use of Laboratory Animals of the National Institutes of Health. All procedures and experiments using mice were designed to use the fewest number of mice possible but still achieve meaningful results. For intravenous inoculations, mice were anesthetized by inhalation of isoflurane to facilitate the procedure and minimize distress. All mice were given food and water ad libitum and were monitored twice daily. Any mice that appeared moribund (e.g. ruffled fur, hunched posture, lack of activity) were euthanized immediately. In any case, euthanasia was performed by inhalation of carbon dioxide, a method consistent with the recommendations of the Panel on Euthanasia of the American Veterinary Medical Association.

### Bacteria

*S*. Typhimurium strain IR715, which is a spontaneous, nalidixic acid-resistant derivative of *S*. Typhimurium strain 14028 (American Type Culture Collection), was used as the wild-type strain. Where indicated, isogenic, *invA*-deficient or enhanced GFP-expressing *S*. Typhimurium were used. These strains were generated by bacteriophage P22-mediated transduction, moving the *invA*::*cam* and *rpsM*::*egfp(kan)* mutations described previously [34], [35] from isogenic derivatives of *S*. Typhimurium strain 14028 into *S*. Typhimurium strain IR715. Bacteria were grown aerobically for 16–18 h at 37°C in 3 ml of Luria-Bertani (LB) broth (250 rpm) or on LB agar using standard microbiological techniques.

### Mice

C57BL/6J mice (8 to 12 weeks of age), which lack a functional *Nramp1/Slc11a1* locus, were purchased from The Jackson Laboratory and used as the wild-type strain of mouse. The *Nramp1/Slc11a1* locus encodes the natural resistance-associated macrophage protein 1 (Nramp1) (Slc11a1) divalent metal transporter, which enhances host resistance to a number of intracellular pathogens, including *S*. Typhimurium, by limiting essential metal availability within the phagocyte phagosome [36]. C57BL/6J mice are phenotypically Nramp1^−^ because of a G169D mutation and have been used as model hosts to study acute salmonellosis. A breeder pair of transgenic C57BL/6J *Nramp1^G169^* mice [36] was generously provided by Dr. Ferric Fang (University of Washington). These mice, which are phenotypically Nramp1^+^, have been used as model hosts to study persistent salmonellosis [37] and were bred at Stony Brook University, Division of Laboratory Animal Resources. C57BL/6J *Nramp1^G169^* mice (8 to 12 weeks of age) bred at Stony Brook University, Division of Laboratory Animal Resources were used for the experiments described in this study.

### Mouse Injections

Mouse injections were performed using naïve, 8- to 12-week-old sex-matched C57BL/6J mice. Briefly, mice were injected intraperitoneally with purified *S*. Typhimurium lipopolysaccharide (LPS) (Enzo Life Sciences) (5 mg/kg) every other day for 10 days. One day after the last injection, pancreata were harvested and processed for analysis by histopathology or flow cytometry.

### Mouse Infections

Mouse infections were performed using naïve, 8- to 12-week-old sex-matched C57BL/6J *Nramp1^G169^* mice. Briefly, mice were inoculated intravenously with 5×10^3^ colony forming units (CFU) of *S*. Typhimurium strain IR715 suspended in 0.1 ml of PBS, unless indicated otherwise. Ten-fold serial dilutions of the inoculum were plated on LB agar to confirm the inoculum titer. At indicated times after inoculation, target organs (i.e. pancreas, liver, and spleen) were harvested and processed for analysis by histopathology, flow cytometry or organ burden assay. Bacterial loads were determined by lysing cells from a single cell suspension with Triton X-100 (0.05%) and plating for CFU on LB agar containing nalidixic acid (50 μg/ml). Mice infected with *S*. Typhimurium were euthanized when moribund or at the termination of the experiment.

### Histological Staining and Quantitation

Pancreatic tissues were fixed overnight in 4% paraformaldehyde, dehydrated and paraffin embedded. Routinely, tissue sections were stained using hematoxylin and eosin (H&E) for overall tissue structure, cytokeratin 19 (Abcam, Cambridge, MA) for metaplasia, and Picrosirius Red Stain Kit (Polysciences) for fibrosis. Immunohistochemistry (IHC) was performed as described previously [38]. Briefly, tissue sections were stained using anti-mouse antibodies specific for collagen I, F4/80 or Ly6B.2 (all from AbD Serotec, Kidlington, UK). Stained tissue sections were examined and photographed using an Olympus BX41 light microscope (Olympus). Inflammatory cell infiltration was quantified by examining 5 representative slides (eight 20x fields per slide) per mouse (n = 4 per group) using ImageScope v11.1.2.752 software (Aperio, Vista, CA). F4/80 and collagen staining was quantified by determining the percentage of positive pixels per field, whereas Ly6B.2 staining was quantified by using an algorithm that calculated positive cell nuclei.

### Fluorescence Microscopy

Pancreatic tissues were fixed for three hours in 4% paraformaldehyde, washed three times (5 minutes per wash) with PBS (0.1 M) and floated overnight in 30% sucrose. Tissues were then incubated for 30 minutes in a 1∶1 mixture of 30% sucrose and optimal cutting temperature compound (OCT), embedded in OCT and frozen at −80°C. Tissue sections of 7 μm each were produced, permeabilized with 0.1% Triton X-100 in 10 mM PBS and blocked with 5% normal donkey serum and 1% BSA in 10 mM PBS for 1 hour at room temperature. Tissue sections were then stained with Alexa Fluor 594 phalloidin (Invitrogen) in 10 mM PBS supplemented with 1% BSA and 0.1% Triton X-100 for 1 hour at room temperature, washed three times with 0.1% Triton X-100 in PBS and rinsed with deionized water. Slides were mounted in VECTASHIELD mounting medium with DAPI (Vector Laboratories). Stained tissue sections were examined and photographed using a Zeiss 510LS Meta confocal microscope (Carl Zeiss MicroImaging).

### Cell Staining and Analysis by Flow Cytometry

Conjugated monoclonal antibodies and reagents described in this section were purchased from BioLegend. Routinely, cells were stained in the presence of Fc block (anti-mouse CD16/32 antibody; clone 93) using anti-mouse antibodies specific for CD11b (clone M1/70), F4/80 (clone CI:A3–1), Ly6C (clone HK1.4) and Ly6G (clone 1A8). Data were acquired and analyzed using a BD FACSCalibur flow cytometer (BD Biosciences) with BD CellQuestPro (BD Biosciences) and FlowJo (Tree Star) software or a BD FACScan flow cytometer (BD Biosciences) with Digital Extra Parameter upgrade (Cytek) and FlowJo Collectors’ Edition software (Cytek).

### Acinar Cell Culture and Infection Assay

The murine acinar cell line 266-6 (American Type Culture Collection) was maintained in DMEM supplemented with 10% fetal bovine serum (Atlanta Biologicals) and sodium pyruvate (1 mM), and incubated at 37°C in 5% CO_2_. Acinar cell infections were performed using a standard gentamicin protection assay [31], [39]. Briefly, acinar cells were suspended in medium lacking antibiotics and seeded at 5×10^5^ cells per ml per well into a 24-well tissue culture plate. After overnight incubation, the medium was replaced with 0.5 ml of fresh medium and the cells were infected with bacteria at a multiplicity of infection of 50. Upon addition of bacteria, the plate was centrifuged for 5 minutes at 1,000 rpm to facilitate bacterial contact with the acinar cells. After 20 minutes of incubation, the wells were washed three times with 1 ml of PBS to remove non-cell-associated bacteria, and fresh medium supplemented with gentamicin (25 μg/ml) was added to each well to kill all extracellular bacteria. This was referred to as the 0 hour time point. After 1 hour of incubation, the wells were washed three times with 1 ml of PBS and the acinar cells were lysed using 0.5 ml of Triton X-100 (0.1%) to release intracellular bacteria. These bacteria were enumerated by plating onto LB agar. In experiments where the acinar cells were infected with GFP-expressing bacteria, the cells were harvested and analyzed by flow cytometry.

### Statistical Analysis

Statistical analysis was performed using Prism 5.0b (GraphPad Software). Data were analyzed using a two-tailed, paired Student’s t-test, or one-way analysis of variance (ANOVA) with Bonferroni’s multiple comparisons posttest; p values<0.05 were considered to be statistically significant. Asterisks indicate statistically significant differences (***p<0.001, **p<0.01, *p<0.05).

## Results

### *S.* Typhimurium LPS Induces Pancreatic Inflammation

**Figure 1:**
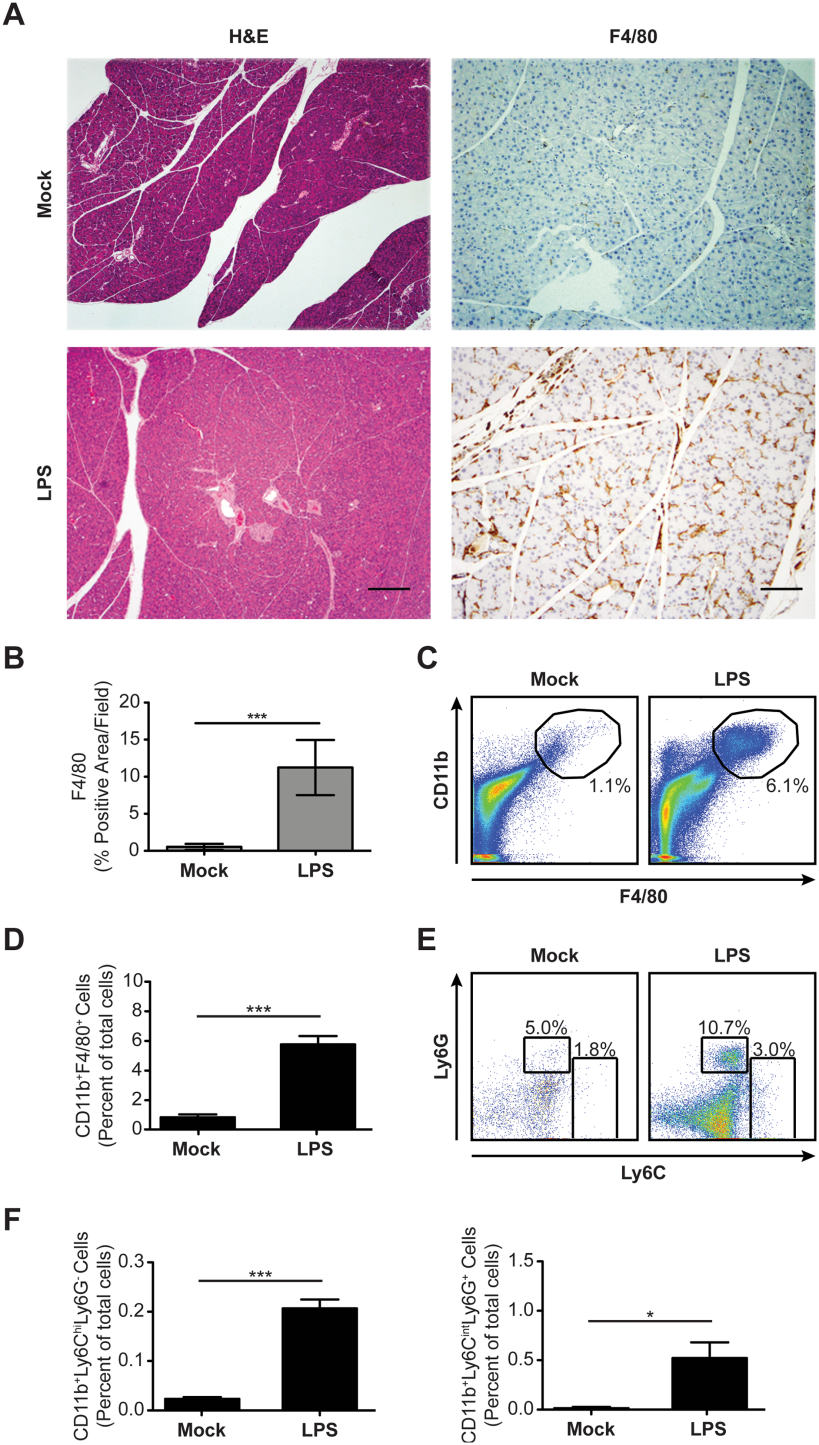
*S*. Typhimurium LPS induces pancreatic inflammation. (*A*) Histological analysis of pancreatic tissue sections from mock-treated or LPS-treated C57BL/6J mice (n = 4 per group). Tissue sections were stained using H&E or subjected to IHC using antibodies specific for F4/80. Scale bars for H&E = 200 μm and for IHC = 100 μm. (*B*) Quantitation of IHC data shown in (A). (*C and D*) Expression of surface F4/80 and CD11b by cells harvested from pancreata of mock-treated or LPS-treated C57BL/6J mice (n = 4 per group) as measured using flow cytometry. Numbers in (C) refer to CD11b^+^ F4/80^+^ cells as percentages of the total numbers of cells. (*E and F*) Expression of surface Ly6C and Ly6G by CD11b^+^ cells present in pancreata of mock-treated or LPS-treated C57BL/6J mice (n = 4 per group) as measured using flow cytometry. Numbers in (E) refer to CD11b^+^ Ly6C^hi^ Ly6G^−^ and CD11b^+^ Ly6C^int^ Ly6G^+^ cells as percentages of the total numbers of CD11b^+^ cells. Data are representative of (A, C, and E), or show mean with SEM from (B, D and F), two independent experiments. Data were analyzed using a two-tailed, paired Student’s t-test; p values<0.05 were considered to be statistically significant. Asterisks indicate statistically significant differences (***p<0.001, *p<0.05).

Bacterial infection, including that caused by *Salmonellae*, has been implicated in human cases of pancreatitis [13], [17]–[27]. Consistent with this notion, *Escherichia coli* LPS exacerbates pancreatitis in alcohol and caerulein-induced animal models, hypothetically through its direct effects on pancreatic acinar cells [40]–[42]. Yet, *Escherichia coli* LPS alone fails to induce the desmoplastic reaction or obvious acinar cell stresses that are hallmarks of pancreatitis [43]. It is generally known that administration of LPS induces a systemic inflammatory response that affects many organs. To determine how the pancreas may be affected by *S*. Typhimurium LPS-induced systemic inflammation, C57BL/6J mice were injected intraperitoneally with *S*. Typhimurium LPS every other day for 10 days. One day after the last injection, pancreata were harvested and processed for analysis by histopathology. Consistent with previous studies [43], H&E staining revealed little tissue damage or associated edema (**Figure 1A**). However, IHC detection of F4/80 and Ly6B.2 revealed a uniform, pancreas-wide distribution of F4/80^+^ macrophages (**Figures 1A and 1B**) with very few Ly6B.2^+^ neutrophils (data not shown). Flow cytometric analysis confirmed the presence of large numbers of F4/80^+^ cells in pancreata of LPS-treated mice as compared to mock-treated mice (**Figure 1C**). These cells also expressed surface CD11b (**Figures 1C and 1D**). The CD11b^+^ cells present in pancreata of mice treated with LPS were a heterogeneous population that consisted mostly of CD11b^+^ F4/80^+^ macrophages (**Figures 1C and 1D**), but also included CD11b^+^ Ly6C^hi^ Ly6G^−^ inflammatory monocytes and CD11b^+^ Ly6C^int^ Ly6G^+^ neutrophilic granulocytes (**Figures 1E and 1F**). Thus, *S*. Typhimurium LPS induces pancreatic inflammation, but fails to induce the reactive epithelial and fibrotic responses that are characteristic of pancreatitis.

### *S.* Typhimurium Infection Induces Pancreatitis

**Figure 2:**
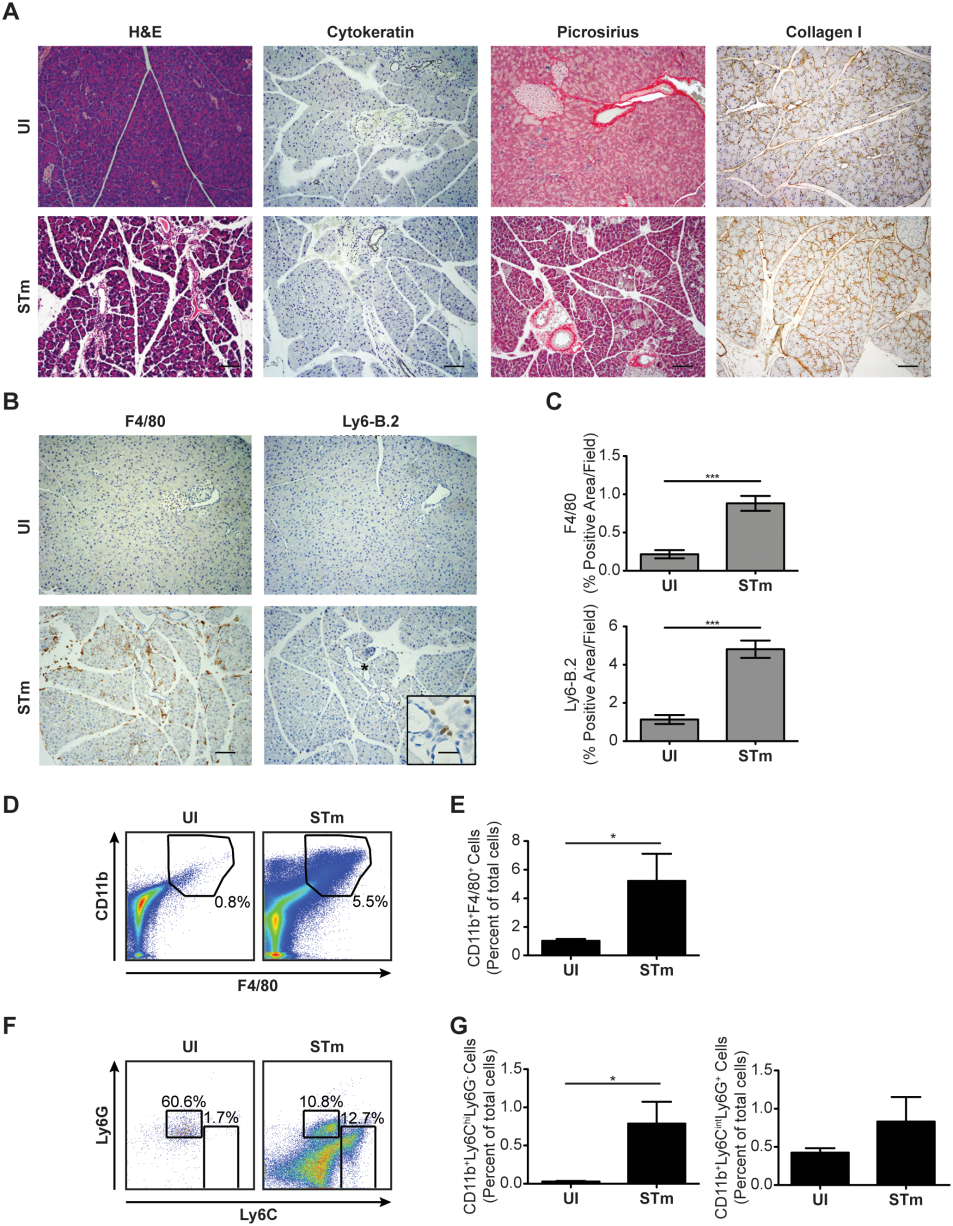
*S*. Typhimurium infection induces pancreatitis. (*A and B*) Histological analysis of pancreatic tissue sections from C57BL/6J *Nramp1^G169^* mice (n = 3–4 per group) left uninfected or infected for 10 days with *S*. Typhimurium (STm). Tissue sections were stained using H&E, cytokeratin 19, or Picrosirius Red Stain Kit. In addition, tissue sections were subjected to IHC using antibodies specific for collagen I (A) or F4/80 or Ly6B.2 (B). Scale bars for H&E = 200 μm and for IHC = 100 μm. (*C*) Quantitation of IHC data shown in (B). (*D and E*) Expression of surface F4/80 and CD11b by cells harvested from pancreata of C57BL/6J *Nramp1^G169^* mice (n = 3–4 per group) left uninfected or infected for 10 days with STm as measured using flow cytometry. Numbers in (D) refer to CD11b^+^ F4/80^+^ cells as percentages of the total numbers of cells. (*F and G*) Expression of surface Ly6C and Ly6G by CD11b^+^ cells present in pancreata of C57BL/6J *Nramp1^G169^* mice (n = 3–4 per group) left uninfected or infected for 10 days with STm as measured using flow cytometry. Numbers in (F) refer to CD11b^+^ Ly6C^hi^ Ly6G^−^ and CD11b^+^ Ly6C^int^ Ly6G^+^ cells as percentages of the total numbers of CD11b^+^ cells. Data are representative of (A, B, D and F), or show mean with SEM from (C, E and G), two independent experiments. Data were analyzed using a two-tailed, paired Student’s t-test; p values<0.05 were considered to be statistically significant. Asterisks indicate statistically significant differences (***p<0.001, *p<0.05).

As *S*. Typhimurium LPS alone was insufficient to induce pancreatitis, we sought to determine the ability of *S*. Typhimurium infection to induce a pancreatitis phenotype more similar to the human disease condition. Therefore, C57BL/6J *Nramp1^G169^* mice were inoculated intravenously with 5×10^3^ CFU of *S*. Typhimurium. After 10 days of infection, an early time point used to assess establishment of salmonellosis, histopathologic analysis showed evidence of mild pancreatic edema, but no cytokeratin 19-positive epithelial metaplasia or significant fibrosis (**Figure 2A**). In addition, IHC for F4/80 and Ly6B.2 revealed the presence of significant numbers of focally pooled macrophages and neutrophils, respectively, in pancreata of mice infected with *S*. Typhimurium as compared to mice left uninfected (**Figures 2B and 2C**). Consistent with these results, we found significantly more CD11b^+^ F4/80^+^ macrophages (**Figures 2D and 2E**), CD11b^+^ Ly6C^hi^ Ly6G^−^ inflammatory monocytes and CD11b^+^ Ly6C^int^ Ly6G^+^ neutrophilic granulocytes (**Figures 2F and 2G**) in pancreata of mice infected with *S*. Typhimurium than in pancreata of mice left uninfected. Thus, *S.* Typhimurium infection induces significant pancreatic inflammation without ductal metaplasia or fibrosis, a phenotype that is similar to acute pancreatitis in humans.

### *S*. Typhimurium Colonizes and Persists in the Pancreas, and can Invade Pancreatic Acinar Cells

**Figure 3:**
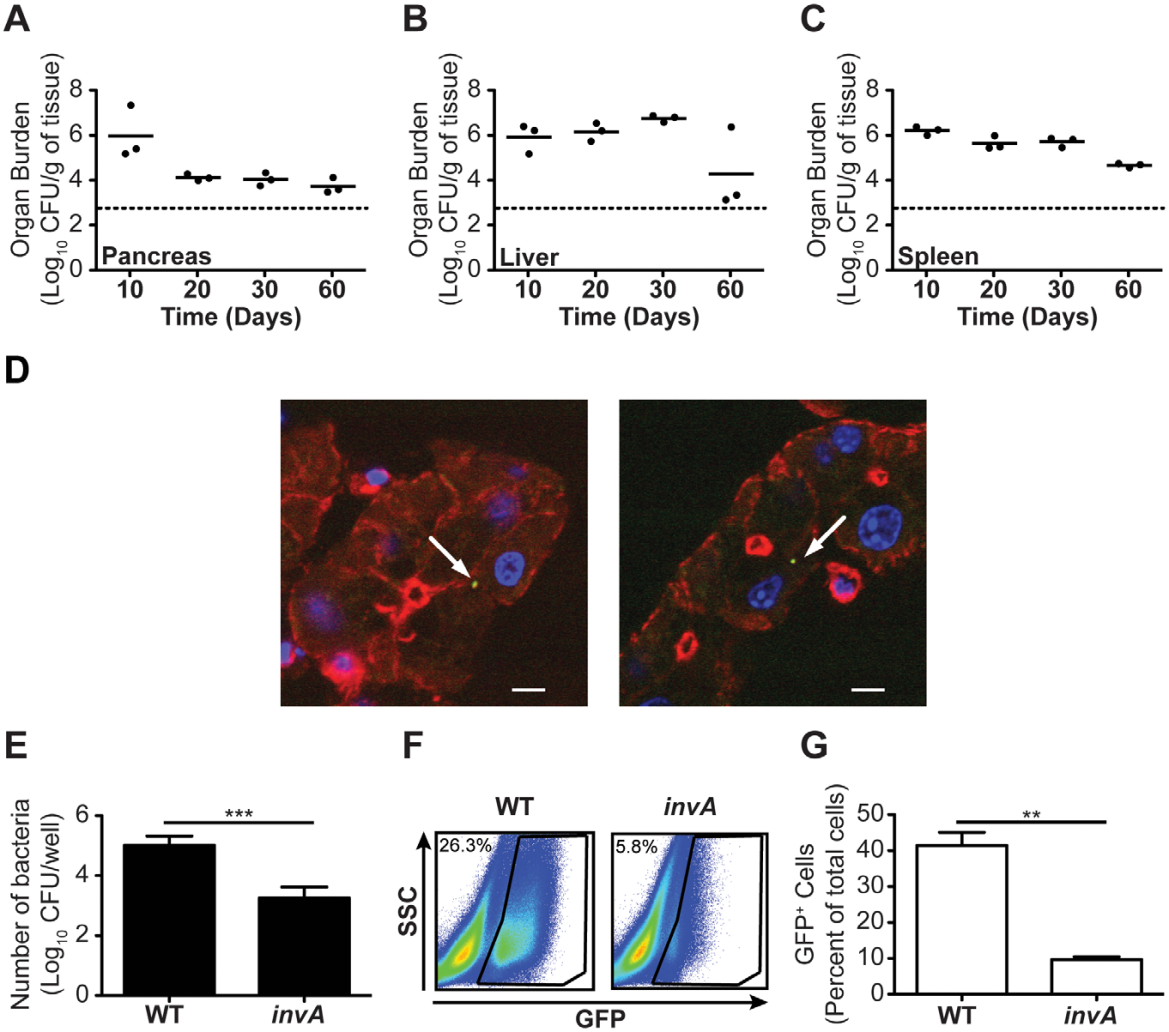
*S*. Typhimurium colonize and persist in the pancreas, associate with pancreatic acinar cells *in vivo*, and can invade pancreatic acinar cells *in vitro*. (*A–C*) Bacterial loads per gram of pancreas (A), liver (B) and spleen (C) tissue harvested from C57BL/6J *Nramp1^G169^* mice (n = 3 per group) at indicated times after infection with *S*. Typhimurium. (*D*) Representative confocal images of pancreatic tissue sections harvested from C57BL/6J *Nramp1^G169^* mice (n = 3 per group) infected with *S*. Typhimurium expressing GFP. Tissue sections were stained with Alexa Fluor 594 phalloidin (red) and DAPI (blue). Arrows point to GFP-expressing *S*. Typhimurium. (*E*) Invasion of cultured pancreatic acinar cells (line 266-6) by wild-type or *invA*-deficient *S*. Typhimurium as measured by gentamicin protection assay. (*F and G*) Detection of GFP associated with cultured pancreatic acinar cells (line 266-6) infected with wild-type or *invA*-deficient *S*. Typhimurium expressing GFP. Data shown in (A–D) show mean with spread from (A–C), or are representative of (D), two independent experiments. Data shown in (E–G) show mean with SEM from (E and G), or are representative of (F), four independent experiments. Data in (E and G) were analyzed using a two-tailed, paired Student’s t-test; p values<0.05 were considered to be statistically significant. Asterisks indicate statistically significant differences (***p<0.001, **p<0.01).

A recent study reported on the detection of *Salmonella* Enteritidis genomic DNA in pancreata of mice infected with *Salmonella* Enteritidis [44], suggesting that *Salmonellae* may colonize the pancreas. To characterize the ability of *S*. Typhimurium to colonize and persist in pancreas, we determined bacterial loads in pancreata of C57BL/6 *Nramp1^G169^* mice inoculated intravenously with 5×10^3^ CFU of *S*. Typhimurium. After 10 days of infection, we recovered substantial numbers of *S*. Typhimurium from pancreata of infected mice (**Figure 3A**). These numbers persisted over a period of 60 days (**Figure 3A**) and were similar to the numbers of *S*. Typhimurium recovered from liver and spleen (**Figures 3B and 3C**). To visualize *S*. Typhimurium in the pancreas, we inoculated C57BL/6 *Nramp1^G169^* mice intravenously with 5×10^3^ CFU of *S*. Typhimurium expressing GFP. After 10 days of infection, we found *S*. Typhimurium associated with acinar cells throughout the pancreas (**Figure 3D**), suggesting that *S.* Typhimurium may directly infect these cells *in vivo*.

To characterize the ability of *S*. Typhimurium to invade pancreatic acinar cells, we infected cultured murine pancreatic acinar cells (line 266-6) with wild-type or *invA*-deficient *S*. Typhimurium at a multiplicity of infection of 50. The *invA* gene encodes an essential structural component of the *Salmonella* Pathogenicity Island (SPI)-1-encoded Type Three Secretion System (TTSS), which is required for invasion of non-phagocytic cells [45]. After 1 hour of infection, we recovered substantial numbers of intracellular bacteria (**Figure 3E**), indicating that *S*. Typhimurium had invaded the acinar cells. We recovered significantly fewer intracellular *invA*-deficient *S*. Typhimurium than wild-type *S*. Typhimurium, indicating that efficient invasion of the acinar cells was dependent on the SPI-1-encoded TTSS (**Figure 3E**). Similar results were obtained when we analyzed by flow cytometry GFP fluorescence of acinar cells infected with wild-type or *invA*-deficient *S*. Typhimurium expressing GFP (**Figures 3F and 3G**). Collectively, these results indicate that *S*. Typhimurium colonize and persist in pancreas, and that the bacterial burden in the pancreas may be due, at least in part, to direct infection of acinar cells.

### Pancreatitis Progresses with Persistent *S.* Typhimurium Infection

**Figure 4:**
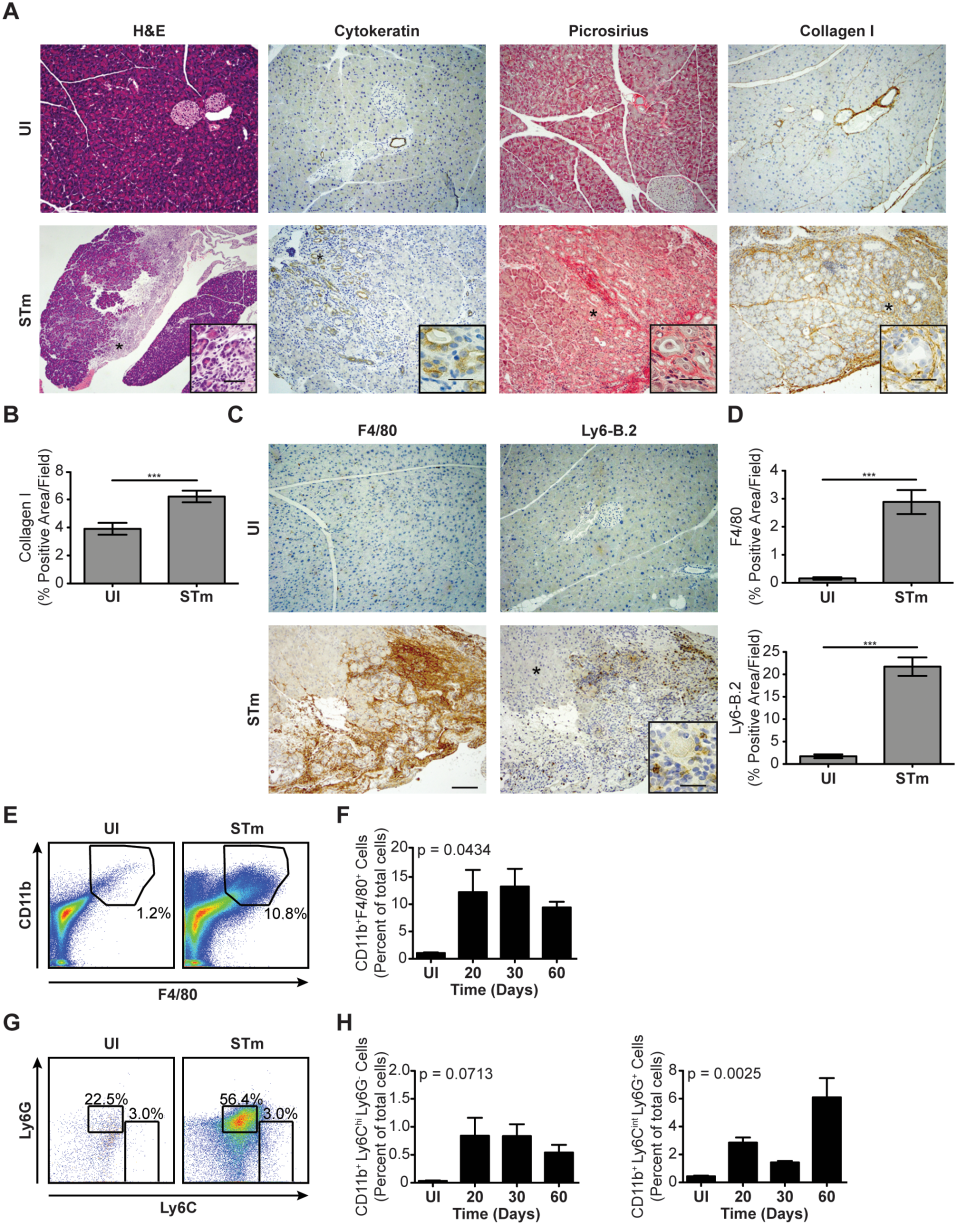
Pancreatitis progresses with persistent *S*. Typhimurium infection. (*A and C*) Histological analysis of pancreatic tissue sections from C57BL/6J *Nramp1^G169^* mice (n = 3–4 per group) left uninfected or infected for 60 days with *S*. Typhimurium (STm). Tissue sections were stained using H&E, cytokeratin 19, or Picrosirius Red Stain Kit. In addition, tissue sections were subjected to IHC using antibodies specific for collagen I (A) or F4/80 or Ly6B.2 (C). Scale bars for H&E = 200 μm and for IHC = 100 μm. (*B and D*) Quantitation of IHC data shown in (A and C). (*E and F*) Expression of surface F4/80 and CD11b by cells harvested from pancreata of C57BL/6J *Nramp1^G169^* mice (n = 3–4 per group) left uninfected or infected for 60 days with STm as measured using flow cytometry. Numbers in (E) refer to CD11b^+^ F4/80^+^ cells as percentages of the total numbers of cells. (*G and H*) Expression of surface Ly6C and Ly6G by CD11b^+^ cells present in pancreata of C57BL/6J *Nramp1^G169^* mice (n = 3–4 per group) left uninfected or infected for 60 days with STm as measured using flow cytometry. Numbers in (G) refer to CD11b^+^ Ly6C^hi^ Ly6G^−^ and CD11b^+^ Ly6C^int^ Ly6G^+^ cells as percentages of the total numbers of CD11b^+^ cells. Data are representative of (A, C, E and G), or show mean with SEM from (B, D, F and H), two independent experiments. Data were analyzed using a two-tailed, paired Student’s t-test (B and D) or a one-way ANOVA (F and H); p values<0.05 were considered to be statistically significant. Asterisks indicate statistically significant differences (***p<0.001).

Given that *S.* Typhimurium induced a pancreatitis-like phenotype during early stages of infection (**Figure 2**) and colonized and persisted in the pancreas over a period of 60 days (**Figure 3**), we next examined pancreata of C57BL/6J *Nramp1^G169^* mice persistently infected with *S*. Typhimurium. Unlike the mild response found after 10 days of *S*. Typhimurium infection (**Figure 2**), we found significant pancreatic damage after 60 days of infection, as indicated by large areas of acinar cell loss made evident by H&E staining (**Figure 4A**). These areas were marked by cytokeratin 19-positive acinar to ductal metaplasia (ADM) and a dramatic desmoplastic response highlighted by substantial collagen deposition (**Figures 4A and 4B**). In addition, the relatively mild inflammatory response induced during early stages of *S*. Typhimurium infection had been replaced by large swaths of F4/80^+^ macrophages and Ly6B.2^+^ neutrophils occupying the damaged areas of the pancreas (**Figures 4C and 4D**). Consistent with these results, we found significantly more CD11b^+^ cells, including CD11b^+^ F4/80^+^ cells (**Figures 4E and 4F**) and CD11b^+^ Ly6C^int^ Ly6G^+^ cells (**Figures 4G and H, right panel**) in pancreata of mice persistently infected with *S*. Typhimurium than in pancreata of mice left uninfected. A similar trend was observed for CD11b^+^ Ly6C^hi^ Ly6G^−^ cells (**Figures 4G and 4H, left panel**), but differences did not reach statistical significance. Similar results were obtained when we examined pancreata of 129×1/SvJ mice persistently infected with *S*. Typhimurium administered intragastrically (data not shown). Collectively, the robust inflammatory response and reactive epithelial and fibrotic responses in the pancreas indicate that persistent *S*. Typhimurium infection induces a progressive condition that is highly similar to chronic pancreatitis in humans.

## Discussion

The etiology of pancreatitis, a known risk factor for the development of PDA, is most commonly associated with alcohol abuse and gallstone-mediated ductal obstruction [1], though frequently the specific cause is unknown. Bacterial infection has frequently been associated with pancreatitis, but is usually considered a consequence of the disease rather than a contributor to the disease. In fact, infection of the pancreas is the primary cause of pancreatitis-associated death [46]. Here, we found that persistent salmonellosis could cause pancreatitis in a murine model of infection. Specifically, we found that pancreatitis induced by persistent *S*. Typhimurium infection was characterized by a loss of pancreatic acinar cells, acinar to ductal metaplasia, fibrosis, and accumulation of inflammatory cells (Figures 2 and 4). Furthermore, we found that *S.* Typhimurium colonized and persisted in the pancreas, associated with pancreatic acinar cells *in vivo*, and could invade cultured pancreatic acinar cells *in vitro* (Figure 3). An immediate implication of our results is that persistent, chronic or repeated infections with *Salmonellae* could lead to the development of pancreatitis.

In humans, *S*. Typhi is the major cause of persistent or chronic salmonellosis. It is estimated that 3–5% of patients infected with *S*. Typhi become chronic carriers. Chronic infections with *S*. Typhi can persist for decades and are often asymptomatic, which makes the identification of chronic carriers difficult. The chronic carrier state has been associated with pre-existing hepatobiliary disease such as the presence of gallstones [47]. Chronic carriers have an increased risk of developing hepatobiliary and pancreatic carcinomas [48]–[53], which links persistent salmonellosis to gastrointestinal cancer. It is generally known that *Salmonellae* can colonize the liver and form biofilms on gallstones in the gallbladder, indicating that direct colonization of these sites may be a mechanism for chronic inflammation, tissue disturbance, and the promotion of a pro-tumorigenic microenvironment [54]. Similarly, our results indicate that direct colonization of the pancreas by *Salmonellae* can cause pancreatic inflammation and tissue injury that is characterized by, but not limited to, metaplasia, a known precursor for neoplastic transformation [55].

To the best of our knowledge, this report is the first to document that an important long-term consequence of persistent salmonellosis may be induction of pancreatitis, a known risk factor for the development of PDA. The similarities between said long-term consequence of persistent salmonellosis and the long-term consequences of chronic infections with *Helicobacter pylori*, a bacterial pathogen that has been identified as one of the primary instigators of intestinal metaplasia in the stomach, are striking. Gastric mucosal tissue injury caused by chronic *H. pylori* infection leads to intestinal metaplasia, which is believed to result from the differentiation of gastric stem cells towards cells of an intestinal phenotype [56]. This intestinal metaplasia carries a significantly increased risk of developing gastric cancer, the second most common cancer globally [57], [58]. Analogously, pancreatic metaplasia due to persistent salmonellosis likely carries an increased risk of developing PDA.

Central to the development of PDA are activating oncogenic *K-ras* mutations [59]. Although *K-ras* mutations alone may not cause PDA, *K-ras* mutations in conjunction with pancreatitis have been shown to induce progression of pancreatic cancer [60]. In addition to tissue injury, we found that pancreatitis induced by *S*. Typhimurium was characterized by a robust inflammatory response. This response consisted of an influx of macrophages, CD11b^+^ Ly6C^hi^ Ly6G^−^ inflammatory monocytes and CD11b^+^ Ly6C^int^ Ly6G^+^ neutrophilic granulocytes (Figures 2 and 4). Recent studies have shown that macrophages, through the secretion of cytokines, can induce pancreatic metaplasia [61]. Furthermore, the CD11b^+^ Ly6C^hi^ Ly6G^−^ and CD11b^+^ Ly6C^int^ Ly6G^+^ cells that accumulate and persist in tissues of mice infected with *S*. Typhimurium resemble myeloid-derived suppressor cells, which have been associated with immunosuppression in cancer and, more recently, infection [62], [63]. We propose a model where persistent salmonellosis induces pancreatic inflammation and tissue injury that may promote the development of metaplasia, which, in conjunction with an activating *K-ras* mutation, may lead to the development of PDA.

In conclusion, we have shown that persistent salmonellosis causes pancreatitis in a murine model of infection. This model recapitulates the complexity of the human disease and, therefore, may be useful for the study of pancreatitis as it relates to bacterial infection. Increased knowledge of how pathogenic bacteria can cause pancreatitis will provide a more integrated picture of the etiology of the disease and could lead to the development of new therapeutic approaches for treatment and prevention of pancreatitis and PDA.
